# Supplementary material for: An apical protein, Pcr2, is required for persistent movement by the human parasite Toxoplasma gondii
Source: PLoS Pathog. 2022 Aug 22;18(8):e1010776. doi: 10.1371/journal.ppat.1010776 (PMC9436145; doi:10.1371/journal.ppat.1010776)
Supplement: S2 Fig — (PDF) [file ppat.1010776.s010.pdf]

S2 Fig

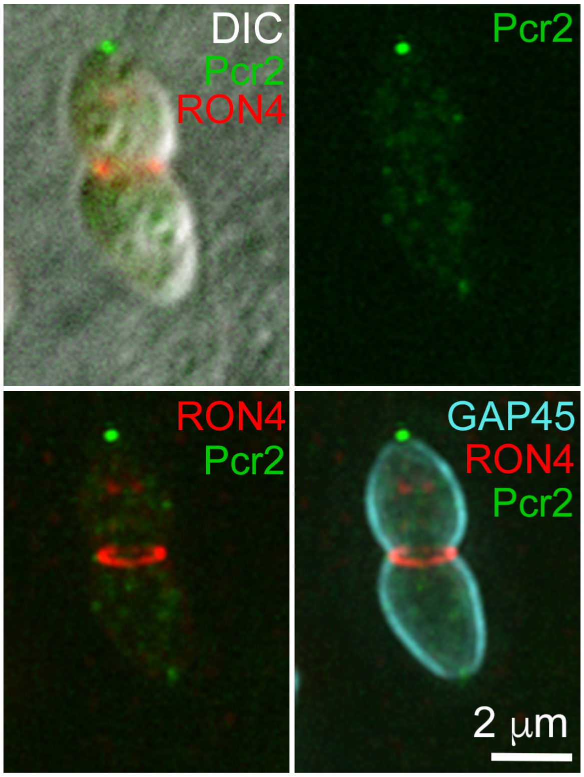

S2 Fig. DIC and projections of deconvolved wide-field fluorescence images of an invading *Pcr2-mNeonGreen* 3' tag parasite (green), in which the moving junction (anti-RON4, red), and the parasite cortex (anti-GAP45, cyan) were labeled by immunofluorescence.
