# Supplementary material for: An apical protein, Pcr2, is required for persistent movement by the human parasite Toxoplasma gondii
Source: PLoS Pathog. 2022 Aug 22;18(8):e1010776. doi: 10.1371/journal.ppat.1010776 (PMC9436145; doi:10.1371/journal.ppat.1010776)
Supplement: S3 Fig — The expression of mEmeraldFP (green) tagged Pcr1 and Pcr3 was driven by a T. gondii tubulin promoter. The parasites were labeled by an anti-IMC1 antibody (red) to highlight the parasite cortex. (PDF) [file ppat.1010776.s011.pdf]

S3 Fig

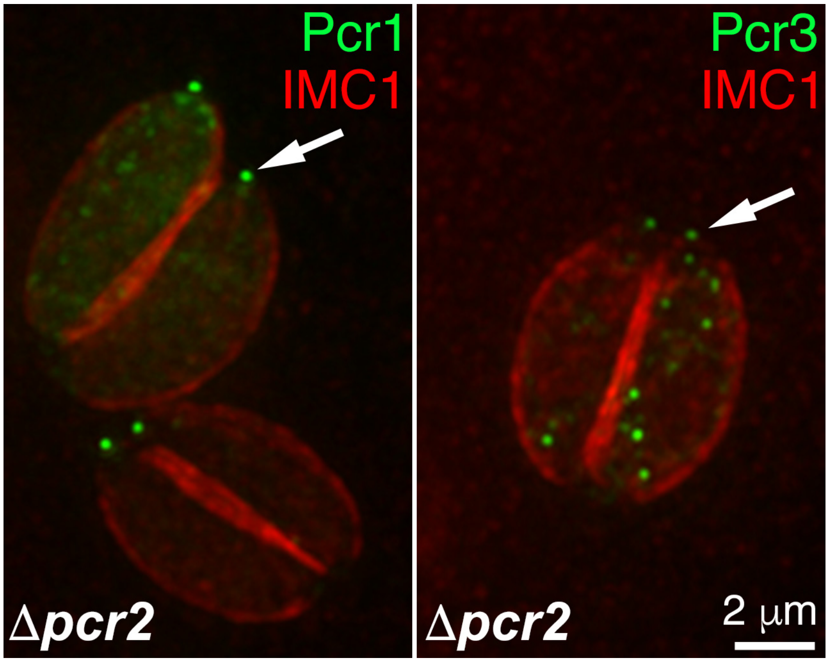

S3 Fig. Ectopically expressed Pcr1 and Pcr3 are found at the apex (arrows) of the *Δpcr2* parasites. The expression of mEmeraldFP (green) tagged Pcr1 and Pcr3 was driven by a *T. gondii* tubulin promoter. The parasites were labeled by an anti-IMC1 antibody (red) to highlight the parasite cortex.
